# The Relationship between Poverty and Healthcare Seeking among Patients Hospitalized with Acute Febrile Illnesses in Chittagong, Bangladesh

**DOI:** 10.1371/journal.pone.0152965

**Published:** 2016-04-07

**Authors:** M. Trent Herdman, Richard James Maude, Md. Safiqul Chowdhury, Hugh W. F. Kingston, Atthanee Jeeyapant, Rasheda Samad, Rezaul Karim, Arjen M. Dondorp, Md. Amir Hossain

**Affiliations:** 1 Mahidol-Oxford Tropical Medicine Research Unit, Faculty of Tropical Medicine, Mahidol University, Bangkok, Thailand; 2 University College, University of Oxford, Oxford, United Kingdom; 3 Centre for Tropical Medicine, Nuffield Department of Clinical Medicine, Churchill Hospital, Oxford, United Kingdom; 4 Chittagong Medical College Hospital, Chittagong, Bangladesh; 5 Global Health Division, Menzies School of Health Research and Charles Darwin University, Darwin, Northern Territory, Australia; Johns Hopkins Bloomberg School of Public Health, UNITED STATES

## Abstract

Delays in seeking appropriate healthcare can increase the case fatality of acute febrile illnesses, and circuitous routes of care-seeking can have a catastrophic financial impact upon patients in low-income settings. To investigate the relationship between poverty and pre-hospital delays for patients with acute febrile illnesses, we recruited a cross-sectional, convenience sample of 527 acutely ill adults and children aged over 6 months, with a documented fever ≥38.0°C and symptoms of up to 14 days’ duration, presenting to a tertiary referral hospital in Chittagong, Bangladesh, over the course of one year from September 2011 to September 2012. Participants were classified according to the socioeconomic status of their households, defined by the Oxford Poverty and Human Development Initiative’s multidimensional poverty index (MPI). 51% of participants were classified as multidimensionally poor (MPI>0.33). Median time from onset of any symptoms to arrival at hospital was 22 hours longer for MPI poor adults compared to non-poor adults (123 *vs*. 101 hours) rising to a difference of 26 hours with adjustment in a multivariate regression model (95% confidence interval 7 to 46 hours; P = 0.009). There was no difference in delays for children from poor and non-poor households (97 *vs*. 119 hours; P = 0.394). Case fatality was 5.9% *vs*. 0.8% in poor and non-poor individuals respectively (P = 0.001)—5.1% *vs*. 0.0% for poor and non-poor adults (P = 0.010) and 6.4% *vs*. 1.8% for poor and non-poor children (P = 0.083). Deaths were attributed to central nervous system infection (11), malaria (3), urinary tract infection (2), gastrointestinal infection (1) and undifferentiated sepsis (1). Both poor and non-poor households relied predominantly upon the (often informal) private sector for medical advice before reaching the referral hospital, but MPI poor participants were less likely to have consulted a qualified doctor. Poor participants were more likely to attribute delays in decision-making and travel to a lack of money (P<0.001), and more likely to face catastrophic expenditure of more than 25% of monthly household income (P<0.001). We conclude that multidimensional poverty is associated with greater pre-hospital delays and expenditure in this setting. Closer links between health and development agendas could address these consequences of poverty and streamline access to adequate healthcare.

## Introduction

Bangladesh, in common with most rapidly developing countries, is subject to profound inequities of wealth and health [1–4]. Poverty—the state of multidimensional deprivation in which basic needs cannot be met—is inextricably linked with disease. This relationship is complex and bi-directional: at its worst, it yields a vicious cycle of deprivation leading to disease, and costs of illness leading to further impoverishment [5]. The goals of alleviating poverty and improving global health have become a converging focus of development and public health initiatives nationally and internationally [6–9]. Reducing inequities of wealth and healthcare has an essential role in addressing the burden of many diseases, and quantifying these inequities is a fundamental prerequisite [7].

Acute febrile illness (AFI) accounts for the majority of illness episodes in the Chittagong Division, and for much of the excess burden of disease associated with poverty [10, 11]. In most cases, the aetiology of AFI is unknown at the time of admission to the referral hospital; once hospitalized, characterization of the disease usually remains limited by available diagnostics and cost to clinical impression from symptoms, signs, basic microbiology, and malaria diagnostics. This aetiologic uncertainty is a major challenge to effective clinical care and public health, and necessitates the pragmatic approach of a broad case definition when considering healthcare-seeking behaviour [12].

Prompt and effective treatment of malaria, meningitis, enteric fever, sepsis, and other causes of serious AFI in this setting can save lives and reduce morbidity [13–17]. Hence, identifying and addressing barriers to care is essential. However, there is limited understanding of the socioeconomic risk factors and consequences of AFI. Most reports on the correlation between AFI and poverty have come from community-based surveys, where the majority of illnesses encountered are self-limiting and minimally investigated [10, 18]. In contrast, patients with the most burdensome and best-characterized infections converge upon the in-patient hospital setting, where reports of morbidity and mortality are frequently compiled, but rarely disaggregated by socioeconomic status.

Newly validated tools—with concise and robust parameters of assessment—facilitate assessment of a patient’s exposure to poverty in the context of an acute illness [18, 19]. The multidimensional poverty index (MPI) was developed by the Oxford Poverty and Human Development Initiative (OPHI) with the aim of providing a validated, easily administered, and internationally applicable metric for assessing household deprivation, and steer recommendations to reduce poverty [20]. This index identifies household living standards, education, and chronic health status (defined by nutritional status and exposure to child mortality) as co-existing dimensions of poverty, and links its assessment parameters directly to the priorities of the Millennium Development Goals. The United Nations Development Programme has recently adopted MPI as an international standard for assessment, tracking, and planning of progress in the global fight against poverty [21].

This investigation seeks to complement previous, community-based studies of the socioeconomic background of people with AFI in Bangladesh, by characterizing the subset of patients admitted for acute medical management [10, 11, 22–24]. We report a survey of patients with AFI attending a large referral hospital in Bangladesh, and describe the relationship between poverty and pre-hospital delays.

## Materials and Methods

### Local structure of healthcare and referral network

Chittagong Division is the largest of Bangladesh’s eight administrative Divisions, with a population of approximately 27 million in 2011, at the time of this study [25]. Public-sector healthcare is the responsibility of Union Health Centers (which provide outreach services focused on prevention) and Upazila/Thana Health Complexes. These Government Health Complexes (GHC) are intended to provide a broad range of out-patient services, and have very limited diagnostic facilities (such as rapid diagnostic tests, RDTs); most also support 30–50 in-patient beds under the supervision of a small medical and nursing team. Secondary level services are provided by District Hospitals, with out-patient facilities, 50 to 250 in-patient beds, and limited laboratory and radiographic capabilities. Within the public sector, consultations with healthcare workers are free of charge, but fees for provision of medication and investigations, as well as inpatient care, vary. Health Complexes and District Hospitals both make direct referrals to tertiary referral hospitals such as Chittagong Medical College Hospital (CMCH), where this investigation was undertaken [26, 27]. The true catchment population of CMCH is difficult to define. In addition to formal referrals from public and private secondary level services, a large number of patients are admitted via the Emergency Department after attending on the informal advice of practitioners or by self-referral.

Alongside public sector health facilities, the private sector delivers a large proportion of medical care at all levels, where payment for consultations, investigations, and treatment is usually out-of-pocket. It is estimated that only 10% of all health and family planning consultations occur in the government sector in Bangladesh [28]. Shops and pharmacies sell over-the-counter and prescription medication, and many shopkeepers and pharmacists give informal medical advice. A spectrum of practitioners operates private chambers for fee-based out-patient consultations, including licensed specialists, other officially qualified para-professionals, and ‘village doctors’ who lack formal qualifications but provide allopathic advice and treatment [18, 27, 28]. For the present investigation, we define doctors as the holders of an MBBS (Medical Bachelor/Bachelor of Surgery), LMF (Licentiate of the State Medical Facility), or higher professional qualification. We define Allopathic Practitioners as those who provide allopathic healthcare advice in a private chamber, but who lack MBBS, LMF, or higher qualification (or whose qualification is unknown). Alongside Allopathic Practitioners, healers from homoeopathic, herbalist, Ayurvedic, and spiritual backgrounds also provide health advice and treatment within the private sector, and are here defined separately, as Traditional Healers [10, 22]. In-patient services are also present in the private sector, with numerous private hospitals, concentrated in urban centers.

### Study site

Chittagong Medical College Hospital (CMCH) is the principal public-sector referral hospital for Chittagong Division. Participants were recruited continuously from September 2011 to September 2012. Ethical approval for this study was obtained from the CMCH Ethical Review Committee and the Oxford Tropical Research Ethics Committee.

The hospital has 1,313 beds, and usually operates at much greater than 100% occupancy. Patients were recruited from the three adult general medical wards and one general pediatric ward. Over the study period, a total of 39,077 patients were admitted to the adult medical wards, and 15,514 to the pediatric ward with all clinical presentations; the total number of patients presenting with AFI was not available.

### Screening and recruitment procedures

Informed, written consent was obtained from patients or legally acceptable representatives in all cases. For adults with capacity to give consent to participate, informed, written consent was obtained from the patient directly. For children and adults without capacity to give consent, informed, written consent was obtained on behalf of the patient from the next of kin, caretakers, or guardians.

A team of six medical and pediatric resident junior doctors acted as interviewers for this survey. All interviewers were fluent speakers of Bengali and Chittagonian. Interviewers received training in Good Clinical Practice for Research, interview techniques, and standard operating procedures for recruitment and use of the survey and anthropometric measurement tools. The target sample size of approximately 500 participants over one year was determined based on the estimated capacity of the interviewers to balance study procedures with their full-time clinical duties. Patients admitted with acute febrile illnesses were identified for screening through daily liaison with the clinical teams responsible for ward admissions. Sampling was structured over time by the minimum target of daily recruitment of one adult and one paediatric patient, but on a given day, if multiple patients were eligible, a convenience sample was taken. Interviews and anthropometric measurements were conducted at the bedside with patients. To assist participants with information recall and obtaining heights, weights, and mid upper arm circumferences, other household members were encouraged to remain at the bedside during the interview and contribute information, provided they and the participant gave verbal consent for them to remain.

Participant eligibility was dependent upon consent, an age of greater than six months, a documented fever of greater than or equal to 38.0°C within 48 hours of admission to a medical or pediatric ward, and a history of symptoms of no greater than 14 days’ duration. Patients and household members aged under 18 years were classified as children, in keeping with the United Nations’ definition, to maintain consistent classification for clinical, occupational, and medico-legal purposes. In keeping with the definition used during Demographic and Health Surveys (DHS) data collection, we regarded all people who usually reside and eat together as household members [29].

### Interview survey

Participants completed a face-to-face, interviewer-assisted survey. A pilot survey was undertaken with 60 participants to test questions for clarity and consistency (data not shown). Pilot data are not included in this analysis, as inclusion criteria changed during the pilot phase.

A 15-minute structured interview recorded 59 multiple-choice, yes/no, and short free-text fields, addressing the following variables: patient and household demographics and location, symptom timespan, sources of help and advice, causes of delays, mode of transport, estimated costs associated with the illness prior to arrival at the referral hospital, time lost from work and household duties (by all working household members—patient plus carers), estimated household income, and other household details needed to calculate the Multidimensional Poverty Index (MPI), as described below.

Participants were interviewed within 24 hours of admission if possible, and followed up until discharge from the ward, transfer to another facility, or death, whereupon this outcome was recorded, along with the provisional diagnosis from the clinical team.

### Calculation of poverty and spending indices

Multidimensional Poverty Index (MPI) was calculated for each household according to the algorithms of the OPHI [20]. Participants were classified on the basis of the MPI of their household as MPI poor or MPI non-poor. In brief, ten dichotomous indicators of deprivation were assessed. Six indicators of deprived living standards were weighted as 1/18 of the MPI score: deprivation of electricity, household assets, floor material, cooking fuel, water source, and sanitation. Two indicators of educational deprivation were weighted as 1/4: lack of school enrolment, and lack of overall educational attainment. Two indicators of chronic health deprivation were weighted as 1/4: presence of malnutrition in any household member, and the occurrence of child mortality within living memory. Missing data were treated according to OPHI recommendations, and a poverty score for each household was calculated as the sum of the ten weighted indicators, to give a value between 0.00 and 1.00. Households with an MPI of greater than 0.33 were classified as multidimensionally poor. Intensity of poverty and MPI of the population as a whole were calculated as described by the OPHI [20]. National MPI values for Bangladesh were obtained from the 2014 OPHI Country Briefing, based on data collected in the 2011 DHS [2, 29].

Participants were asked to estimate income in an average month from all sources; this was divided by the number of adults in the household to determine income in Tk per adult equivalent (AE) per month. Adult equivalent income was calculated from the number of adults and children in the household using the OECD Equivalence Scale, as AE = 1+0.7(N_adults_-1)+0.5N_children_ [30]. Households were classified as above or below wealth thresholds calculated from private individual purchasing power parity (PPP), using the World Bank’s PPP analysis in 2010, adjusted to the currency exchange rate from the start of the study period (1347Tk/AE/month for US$1.25/adult/day and 2155Tk/AE/month for US$2.00/adult/day) [31].

Participants were asked to estimate and characterize costs relating to illness incurred up to the point of admission to hospital, and to describe how these costs were met. Estimated total expenditure during the illness episode prior to hospital admission was used to determine if participants had exceeded established definitions of catastrophic expenditure at 25%, 40%, and 100% of total monthly household income [32].

### Sequence of healthcare providers and timecourse estimation

To characterize healthcare-seeking behavior, participants were first asked to narrate the steps taken in seeking help with the illness, listing all sources of help outside of the home, which had been consulted during this illness episode, up to the point of arrival at CMCH. Interviewers then screened for omitted sources from a list of common options. Participants were then asked about the sequence in which these sources were consulted, based on the time of first consultation with each. Repeated consultations with the same provider were scored as a single episode, in keeping with previous studies [33].

Having established the sequence of sources of help, estimates of the timecourse of the illness and healthcare-seeking behavior were sought. Participants estimated the date and time at which the first symptom arose, approximating where a precise time or date could not be recalled by reference to day, night, and mealtimes. Participants estimated three further milestones: the time and date of the decision to seek help outside of the home (from the first source in the sequence of healthcare providers); the time and date of the decision to escalate care by coming to the referral hospital; and the time and date of arrival at the hospital. These milestones were used to calculate total timespan of healthcare-seeking, subdivided into (i) the timespan from onset of symptoms to help outside the home; (ii) timespan from first help to the decision to escalate to CMCH; and (iii) the timespan from this decision to arrival at the hospital.

Participants were questioned about perceived sources of delay in decision-making and transport, screening from a list of common causes, with scope to volunteer additional answers.

### Measurement of height, weight, and mid upper arm circumference

Measurements of standing height, weight, and left mid upper arm circumference (MUAC, cm) were obtained from patients and all available household members. Thresholds for malnutrition were adapted from the OPHI standards used for national calculations of MPI [20]. For children, age- and sex-specific standard distributions were obtained from the World Health Organization, and individuals more than two standard deviations from the mean (on the basis of MUAC for children under five and BMI for those five and over) were classed as malnourished [34, 35]. Adults were classed as malnourished if they had BMIs of less than 18.5. For participants who were unable to stand, MUAC alone was used, with a threshold of two SD below the age- and sex-specific mean for those under 18, <20 cm for men aged 18 or over, and <19 cm for women aged 18 or over [34].

### Data management and analysis

Double data entry was performed using OpenClinica Database Software v.3.1.3, with discrepancies and empty fields prompting review of the original case record forms for clarification and correction. To cross-check accurate ascertainment in the face-to-face survey, the records of 67 participants were validated with telephone follow-up to the participant from a second researcher after discharge from hospital, confirming that key parameters had been correctly ascertained.

Statistical analysis was performed using STATA/IC software v.11.2 (StataCorp, College Station, TX, USA) and Prism v.6.0b (Graphpad Software, La Jolla, CA, USA). Rank correlations between ordinal MPI score (0.00 to 1.00) and other variables were sought using Spearman’s rho in view of the score’s non-Gaussian distribution. For univariate analysis, associations between MPI status (poor or non-poor) and dichotomous variables were sought using proportion tests when all group sizes were greater than 10, and Fisher’s exact test when less than 10. Correlations between MPI status and continuous variables showing a non-Gaussian distribution were sought using the Mann-Whitney U test. The relationship between pre-hospital illness timespan and explanatory variables was interrogated with multiple linear regression analysis using the STATA software package. P-values of <0.05 were considered statistically significant.

## Results

### Demographic and clinical characteristics of the study population

**Fig 1:**
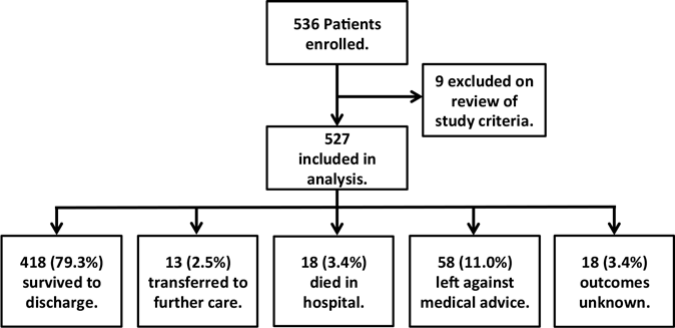
Study participants and admission outcomes, from September 2011 to September 2012. Limited admission record-keeping prevented ascertainment of the total number of eligible patients during the study period.

536 acutely febrile participants were recruited into this study; nine of these were excluded upon review of data for failing to meet inclusion criteria, and data from the remaining 527 are presented for the remainder of this report (**Fig 1**). The study population comprised 242 adults (18 and older) and 285 children; demographic and clinical characteristics are shown in **Table 1**. The age distribution reflects that of the general population of Bangladesh, but shows a greater preponderance of children under five. 49% of recruitment was from the Paediatric Ward, compared to 40% of all hospital admissions during the study period. 63% of participants were male, compared to 53% of all hospital admissions during the study period, and 51% of the general population [36].

**Table 1:** Baseline characteristics of participants and households.

		All	MPI Poor	MPI Non-Poor	Poor *vs*. Non-Poor
Characteristic		*n* = 527	*n* = 269	*n* = 258	P-value*
**Age**	> 0.5 to < 5 years (%)	152 (29%)	92 (34%)	60 (23%)	<0.001
	5 to < 10 years (%)	69 (13%)	46 (17%)	23 (9%)	
	10 to < 18 years (%)	64 (12%)	33 (12%)	31 (12%)	
	18 to < 30 years (%)	126 (24%)	47 (17%)	79 (31%)	
	30 to < 40 years (%)	45 (9%)	14 (5%)	31 (12%)	
	40 to < 50 years (%)	29 (6%)	16 (6%)	13 (5%)	
	50 to < 60 years (%)	18 (3%)	10 (4%)	8 (3%)	
	> 60 years (%)	24 (5%)	11 (4%)	13 (5%)	
**Sex**	Male (%)	330 (63%)	168 (62%)	162 (63%)	0.936
	Female (%)	197 (37%)	101 (38%)	96 (37%)	
Children in Household, median [IQR, range]		2[1–3, 0–45]	2 [2–3, 0–9]	2 [1–3, 0–45]	0.003
Adults in Household, median [IQR, range]		4 [2–5, 1–23]	3 [2–5, 1–13]	4 [3–5, 1–23]	0.006
**Residence**^a^	Rural (%)	296 (56%)	184 (68%)	112 (43%)	<0.001
	Urban (%)	23(44%)	85 (32%)	146 (57%)	
**Distance to CMCH in hr**, median [IQR, range]^b^		2 [1–3, 0.1–13]	2 [1–3, 0.2–10]	1 [0.5–2, 0.1–13]	<0.001
**Length of Stay in days**, median [IQR, range]^c^		5 [4–8, 0–64]	6 [4–8, 1–48]	5 [3–7, 0–64]	0.003
**Mortality**	Overall (%)	18 (3.4%)	16 (5.9%)	2 (0.8%)	0.001
	Among Children *n* = 285 (%)	13 (4.6%)	11 (6.4%)	2 (1.8%)	0.083
	Among Adults *n* = 242 (%)	5 (2.1%)	5 (5.1%)	0 (0%)	0.010

*P-values represent comparisons of all poor *vs*. all non-poor participants. P-values are derived from Wilcoxon Rank Sum test for ordinal variables (age, household size, and length of stay), proportion test was used for dichotomous variables with large group sizes (sex, rural/urban residence), and Fisher’s exact test for mortality in view of small group sizes.

^a^Participants self-categorized households as rural or urban.

^b^From 525 patients;

^c^From 508 patients (deaths excluded).

The diagnostic categories used in the study differed from those routinely collected by the hospital, but patterns of disease within the study appeared to correspond with the disease profile of AFI admissions (unpublished data, MAH). The overall case fatality in the study population was 3.4% (2.1% for adults and 4.6% for children).

### Determination and validation of Multidimensional Poverty Index status

**Fig 2:**
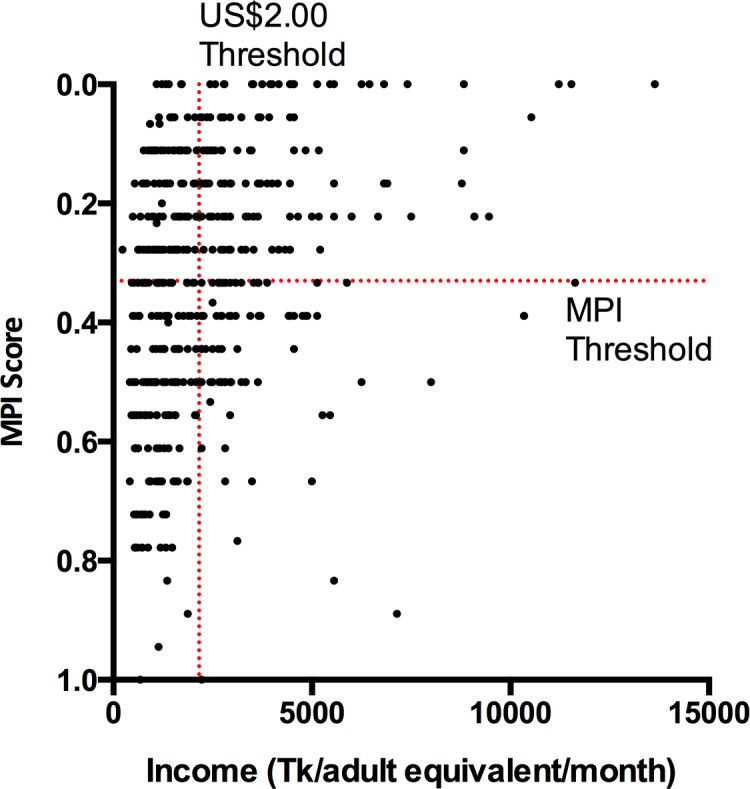
MPI score *vs*. average household income per adult equivalent per month. The correlation is significant with a P-value of <0.001 and a Spearman’s rho of -0.37. Thresholds for MPI and US$2.00 daily income per adult equivalent (adjusted to the World Bank’s private individual purchasing power parity estimate) are shown for reference. 56% of study households fall below the wealth threshold of US$2.00/adult/day, and 51% fall below the MPI threshold for classification as multidimensionally poor.

MPI results and their decomposition are shown in **Table 2**, alongside national statistics from the 2011 DHS dataset [2]. In keeping with findings of the DHS in the Chittagong Division, a large percentage of participants had deprivation of cooking fuel, floor material, household assets, and malnutrition. A smaller percentage of both the study population and national surveys were deprived in terms of access to drinking water, exposure to child mortality, and non-enrollment of school-aged children. **Fig 2**shows that MPI score and average monthly income per adult correlate inversely, with a higher poverty index associated with a lower self-estimated income (P<0.001; Spearman’s rho -0.37). Using the standard MPI threshold of 0.33—indicating substantial deprivation in at least one third of weighted indicators [20]—51% of participants (95%CI 47–55%) came from households classified as multidimensionally poor. 41% of adult patients and 60% of children came from households classed as MPI poor. The intensity of poverty in the study population as a whole (the mean percentage of weighted indices present) was 34% (95%CI 32–35%). Multidimensional poverty index (percentage of population living in poverty x intensity of poverty) of the study population as a whole is 0.171. The 2011 DHS indicates that in Chittagong Division as a whole at the time of the study, 51% of the population were multidimensionally poor (95%CI 46–55%), with an overall intensity of poverty of 50% (95%CI 47–52%) and overall MPI of 0.252 [2].

**Table 2:** Prevalence of MPI components as % of the population, among study participants compared to the DHS 2011 Survey of Chittagong Division.

		StudyParticipants		Chittagong Division (DHS 2011)	
		*n* = 527		*n* = 14,995	
MPI Component^a^		% deprived	(95%CI)	% deprived	(95%CI)
Education:	Schooling	**35.5**	**(31.4, 39.7)**	**17.6**	**(13.9, 21.3)**
	Child School Attendance	**26.5**	**(22.8, 30.5)**	**17.2**	**(13.5, 20.9)**
Health:	Child Mortality	**13.4**	**(10.6, 16.7)**	**25.0**	**(22.5, 27.6)**
	Nutrition	**45.0**	**(41.4, 45.0)**	**35.2**	**(32.1, 38.3)**
Living Standards:	Electricity	**23.3**	**(19.8, 27.2)**	**33.3**	**(26.9, 39.8)**
	Improved Sanitation	**34.5**	**(30.5, 38.8)**	**39.5**	**(34.4, 44.6)**
	Drinking Water	**9.9**	**(7.5, 12.7)**	**1.8**	**(1.0, 2.5)**
	Floor	**44.2**	**(39.9, 48.6)**	**75.2**	**(72.0, 78.3)**
	Cooking Fuel	**75.0**	**(71.1, 78.7)**	**87.6**	**(84.3, 91.0)**
	Asset Ownership	**55.7**	**(51.3, 60.0)**	**83.1**	**(80.4, 85.9)**

^a^ Each component is scored dichotomously with the following criteria: schooling deprived if no member of the household has completed more than five years of full-time education; child school attendance deprived if the household has children aged 5–13 who are not in full-time school; child mortality exposed if, within living memory of the survey participant, any child in the household has died; nutrition deprived if any member of the household available for measurement meets anthropometric criteria for malnutrition; electricity deprived if the household has no electricity; improved sanitation deprived if the household’s toilet does not meet MDG standards, or is shared with other households; drinking water deprived if the household’s usual source of drinking water does not meet the MDG standards for an improved water source, or if that source is more than 30 minutes’ journey away; floor deprived if the floor is made of earth, sand, or dung; cooking fuel deprived if the household cooks with wood, charcoal, dung, straw, shrubs, or grass; asset ownership deprived if the household has no car, truck, or tractor and has fewer than two items from a list of radio, television, telephone, refrigerator, bicycle, and motorcycle.

### Household and disease characteristics disaggregated by MPI status

The demographic characteristics of the study population disaggregated by MPI status are shown in **Table 1.** A larger proportion of participating children under 10 were from MPI poor households, and a larger proportion of young adults were from non-poor households. The disproportionate ratio of male to female patients was observed consistently in both MPI poor and non-poor populations. MPI poor households had more children and fewer adults. A larger proportion of the MPI poor population came from households in a rural setting, at a greater distance from the hospital (with a median journey duration of 2 hours for poor and 1 hour for non-poor households). These observations are consistent with known geographical and demographic patterns of the Chittagong Division [10].

Length of admission was marginally greater for MPI poor participants. 98% of participants across both groups were admitted for at least 48 hours. Case fatality was 5.9% *vs*. 0.8% in poor and non-poor individuals respectively (P = 0.001)—5.1% *vs*. 0.0% for poor and non-poor adults (P = 0.010) and 6.4% *vs*. 1.8% for poor and non-poor children (P = 0.083).

The working diagnoses, recorded at the time of discharge from the ward or death, are summarized in **Table 3**. In most cases, the diagnosis was clinical, as laboratory and radiological services in the hospital are limited. CNS infections and malaria comprised a significantly higher proportion of AFIs in the MPI poor group, which were also the diseases carrying the greatest risk of death. Enteric fever and dengue fever were clinically diagnosed in a significantly greater proportion of the non-poor group.

**Table 3:** Summary of clinical diagnoses and deaths, disaggregated by age and MPI groups.

	All		MPI Poor		MPI Non-Poor		Poor *vs*. Non-Poor
	*n* = 527 (18 deaths)		*n* = 269 (16 deaths)		*n* = 258 (2 deaths)		
Diagnostic Category	*n* (%)	Died	*n* (%)	Died	*n* (%)	Died	P-value^a^
Respiratory Tract Infection	110 (21%)	**.**	56 (21%)	**.**	54 (21%)	**.**	0.351
Central Nervous System Infection	93 (18%)	**11**	61 (23%)	**9**	32 (12%)	**2**	**0.002**
Enteric Fever^b^	78 (15%)	**.**	31 (12%)	**.**	47 (18%)	**.**	**0.037**
Urinary Tract Infection	55 (10%)	**2**	24 (9%)	**2**	31 (12%)	**.**	0.258
Malaria	38 (7%)	**3**	28 (10%)	**3**	10 (4%)	**.**	**0.004**
Dengue Fever^b^	34 (6%)	**.**	10 (4%)	**.**	24 (9%)	**.**	**0.012**
Febrile Convulsion	23 (4%)	**.**	12 (4%)	**.**	11 (4%)	**.**	1.000
Hepatobiliary Infection	23 (4%)	**.**	12 (4%)	**.**	11 (4%)	**.**	1.000
Gastrointestinal Infection	10 (2%)	**1**	7 (3%)	**1**	3 (1%)	**.**	0.340
Sepsis	9 (2%)	**1**	5 (2%)	**1**	4 (2%)	**.**	1.000
Soft Tissue Infection	8 (2%)	**.**	6 (2%)	**.**	2 (1%)	**.**	0.286
Undifferentiated Febrile Illness	46 (9%)	**.**	17 (6%)	**.**	29 (11%)	**.**	0.063

^a^Comparison of diagnostic category incidence for MPI poor *vs*. MPI non-poor participants, by Fisher’s exact test.

^b^Enteric Fever and Dengue Fever were common clinical diagnoses, but could rarely be confirmed by microbiological/virological investigations, due to a lack of laboratory resources.

### Patterns of care-seeking prior to Referral Hospital admission

**Fig 3:**
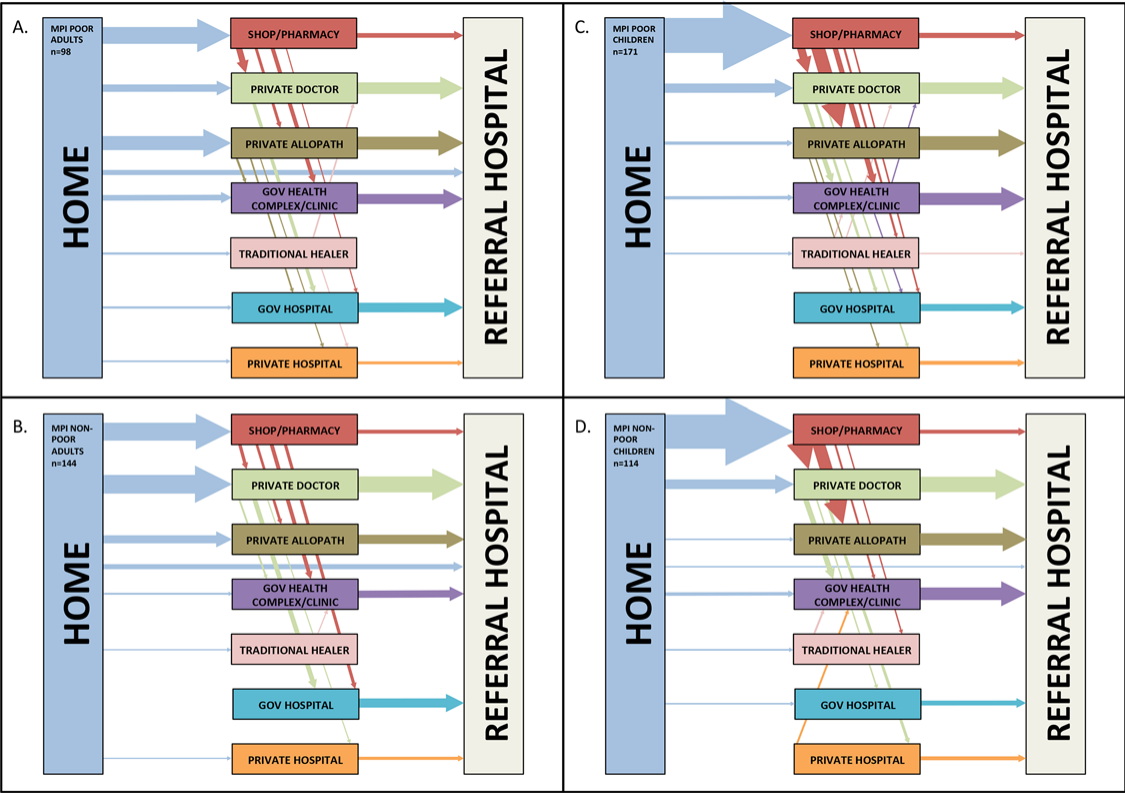
Patterns of care-seeking among participants prior to referral hospital admission. Patterns among MPI poor adults (A.), MPI non-poor adults (B.), MPI poor children (C.) and MPI non-poor children (D.). Arrows represent transitions between providers. Arrow sizes are proportional to the number of participants making a transition between each pair of care providers, and ordered by frequency from top to bottom for the transitions between home and initial source of help, and by frequency from left to right for transitions between sources of help consulted prior to the referral hospital. Transitions undertaken by fewer than 2% of the group are not illustrated.

**Fig 3 and Table 4**summarize the care-seeking practices of participants; a complete record of this transition analysis is included as **S1 and S2 Tables** online. Among adults, participants from poor households were more likely to consult an unqualified allopathic practitioner, whereas participants from non-poor households were more likely to consult a qualified private doctor. Among children, patterns of utilization were similar for poor and non-poor participants. Children from poor and non-poor groups were more likely to seek help from shops/pharmacies than adults.

**Table 4:** Patterns of care-seeking among poor and non-poor adults and children.

**Adult care-seeking pattern**	**All Adults**	**MPI Poor**	**MPI Non-Poor**	**Poor *vs*. Non-Poor**
	***n* = 242**	***n* = 98**	***n* = 144**	
**Source**	***n* (%)**	***n* (%)**	***n* (%)**	**P-value^a^**
Shop/Pharmacy	79 (33%)	32 (33%)	47 (33%)	1.000
Private Doctor	97 (40%)	29 (30%)	68 (47%)	**0.007**
Private Allopath	60 (25%)	31 (32%)	29 (20%)	**0.049**
Government Health Complex/Clinic	45 (19%)	22 (22%)	23 (16%)	0.240
Government Hospital	41 (17%)	17 (17%)	24 (17%)	1.000
Traditional Healer	8 (3%)	4 (4%)	4 (3%)	0.718
Private Hospital	18 (7%)	9 (9%)	9 (6%)	0.458
Friends/Relatives	4 (2%)	. .	4 (3%)	0.149
Other Source	2 (1%)	2 (2%)	. .	.
**Child Care-seeking patterns**	**All Children**	**MPI Poor**	**MPI Non-Poor**	**Poor *vs*. Non-Poor**
	***n* = 285**	***n* = 171**	***n* = 114**	
**Source**	***n* (%)**	***n* (%)**	***n* (%)**	**P-value**^**a**^
Shop/Pharmacy	187 (66%)	113 (66%)	74 (65%)	0.899
Private Doctor	129 (45%)	70 (41%)	59 (52%)	0.089
Private Allopath	85 (30%)	55 (32%)	30 (26%)	0.355
Government Health Complex/Clinic	81 (28%)	48 (28%)	33 (29%)	0.894
Government Hospital	35 (12%)	24 (12%)	11 (10%)	0.357
Traditional Healer	20 (7%)	12 (7%)	8 (7%)	1.000
Private Hospital	27 (9%)	15 (9%)	12 (11%)	0.692
Friends/Relatives	6 (2%)	5 (3%)	1 (1%)	0.407
Other Source	3 (1%)	2 (1%)	1 (1%)	**.**

^a^ Comparison of source among poor and non-poor participants by Fisher’s exact test.

The number of providers consulted was consistent between poor and non-poor participants (median of 2 providers before CMCH for both groups). 57% of poor and 63% of non-poor participants had sought help from two or more providers before attending CMCH (P = 0.254). 19% of poor and 16% of non-poor participants sought help from three or more sources (P = 0.156), and 3% of poor and non-poor had sought help from four or more providers before hospitalization at CMCH.

### Timespan of pre-hospital care-seeking

**Fig 4:**
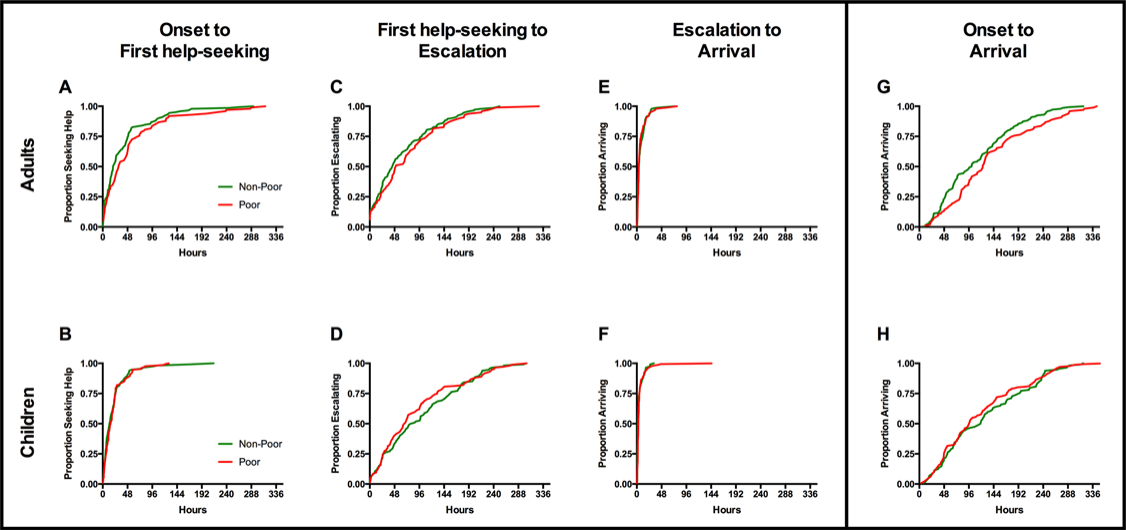
Cumulative histograms of time to key stages in care-seeking. The total timespan from onset of symptoms to arrival at the referral hospital is subdivided into three stages: (A, B) time until the first decision to seek help outside the home; (C, D) time from this decision until escalation to a referral hospital; (E, F) time from the decision to escalate until arrival. Panels G and H represent the cumulative timecourse of all three stages for adults and children respectively.

**Fig 4 Panels A through F** illustrate the time taken to each of the three stages from onset of symptoms to arrival at CMCH for adults and children, and **panels G and H** show cumulative timecourse from the complete duration of illness from onset of symptoms to arrival at CMCH. Wide variation was observed in the total time from onset of symptoms to arrival at the referral hospital.

The majority of participants sought help outside of the home soon after the onset of symptoms. Among adults, MPI poor participants took a median of 10 hours longer than non-poor to decide to seek help outside the home (31 hours from onset for poor *vs*. 21 hours for non-poor; P = 0.049). The difference between poor and non-poor adults became more pronounced for the decision to escalate care to the referral hospital, with a difference in median time of 22 hours (118 hours for poor *vs*. 96 hours for non-poor from onset to decision to escalate; P = 0.032). Once the decision to attend CMCH was made, few participants experienced major delays in transport, and these delays affected poor and non-poor participants similarly, such that median total times from onset of symptoms to arrival at CMCH of 123 hours for poor adults *vs*. 101 hours for non-poor (P = 0.021).

Comparing children from poor and non-poor households, we found no evidence of difference in care-seeking timecourses. Median times to first seeking help outside the home were 14 and 16 hours for children from poor and non-poor households respectively, with no evidence of difference (P = 0.749). Median times until escalation to the referral hospital were 93 hours from onset for poor and 111 hours for non-poor children (P = 0.267), and median times to arrival were 97 and 119 hours for poor and non-poor children respectively (P = 0.395).

Using a multiple regression model, we analyzed demographic or geographical differences between the MPI poor and non-poor groups to explain observed differences in referral time. In addition to MPI status the following variables were included in the model: sex, age, and journey duration to hospital. Urban or rural classification was considered for the model, but dropped because of its strong correlation with distance to hospital. Pairwise correlation between the remaining variables confirmed no multicollinearity. In this model, there was no significant interaction of MPI status and distance to hospital.

**Table 5**summarizes the multiple linear regression models for adults, children, and the combined population. For adults, MPI poor was associated with greater pre-hospital delays, independent of the other variables included in the model. The model indicates that, across the population of adults with AFI, MPI poor patients face an additional 26 hours in the timespan from symptom onset to hospital arrival (95% CI: 7 to 46 hours) compared to non-poor patients.

**Table 5:** Multiple linear regression analyses of pre-hospital illness timespan (in hours) for adults, children, and all participants.

	Crude Coefficient (β)^a^	95% CI	P-value	Adjusted Coefficient (β)^a^	95% CI	P-value
***Adults*:**						
**MPI Poor**	**25.5**	**5.7 to 45.2**	**0.012**	**26.5**	**6.6 to 46.3**	**0.009**
**Distance to Hospital (hr)**	**8.9**	**1.9 to 15.9**	**0.013**	**8.4**	**1.5 to 15.4**	**0.017**
Male	14.6	-5.4 to 34.5	0.152	17.8	-1.9 to 37.5	0.077
Age (yr)	-0.5	-1.1 to 0.1	0.128	-0.62	-1.3 to 0.0	0.054
***Children*:**						
MPI Poor	-9.1	-28.5 to 10.2	0.354	-9.6	-28.9 to 9.7	0.327
Distance to Hospital (hr)	4.7	-2.0 to 11.4	0.170	5.9	-0.8 to 12.6	0.085
Male	-3.2	-23.1 to 16.7	0.751	-3.3	-22.8 to 16.2	0.741
**Age (yr)**	**3.9**	**1.9 to 5.9**	**<0.001**	**3.9**	**1.9 to 6.0**	**<0.001**
***Adults and Children*:**						
MPI Poor	6.2	-7.4 to 19.8	0.368	3.5	-10.4 to 17.4	0.625
**Distance to Hospital (hr)**	**6.2**	**1.5 to 10.9**	**0.010**	**6.3**	**1.4 to 11.1**	**0.012**
Male	5.1	-8.9 to 19.1	0.478	6.0	-8.1 to 20.1	0.402
Age (yr)	0.0	-0.4 to 0.4	0.977	0.1	-0.3 to 0.5	0.612

^a^ The coefficient (**β**) reflects the magnitude (in hours) of the effect on the pre-hospital timespan associated with the variable’s presence (in the case of the dichotomous variables MPI poor and male sex) or of each unit of the continuous variables, distance to hospital (in hours) or age (in years). Crude (univariate) and adjusted (multivariate) coefficients are shown for each model. Variables associated with a statistically significant increase in pre-hospital timespan in the multiple linear regression model are set in bold.

For adults, each hour of estimated travel time was associated with an additional 8.4 hours (95%CI: 1.5 to 15.4 hours) added to the total duration of illness before hospitalization. The equivalent model for children yielded age as the only significant predictor of time from onset to arrival, contributing 3.9 hours per year of age (95%CI: 1.9 to 6.0 hours). In the model for adults and children combined, distance from hospital was the only significant predictor of time from onset to arrival, with each hour of travel time associated with 6.3 hours added to the pre-hospital journey (95%CI: 1.4 to 11.1 hours).

### Perceived delays in pre-hospital care

Perceived contributors to delays in reaching hospital are summarized in **Table 6**. Thirteen percent of participants concluded that they had faced no delays in decision-making. Across both MPI groups, the most prevalent perceived delay in decision-making was medical treatment elsewhere, followed by uncertainty as to whether the patient was unwell enough to require hospitalization. These two responses were the only answers to significantly correlate with an increase in total time from onset of symptoms to admission. MPI poor participants were more likely to report delaying the decision to attend the referral hospital because of a lack of money.

**Table 6:** Summary of perceived delays affecting the decision to attend referral hospital, and delays to transport after this decision.

	MPI Poor	MPI Non-Poor	All	Proportion test
	*n =* 269	*n* = 258	*n =* 527	
Perceived delay to decision to come to the hospital	*n* % Of group	*n* % of group	*n* % of group	P-value
Undergoing medical treatment elsewhere	170 (63%)	155 (60%)	325 (62%)	0.462
Unsure if unwell enough	108 (40%)	107 (41%)	215 (41%)	0.757
Not enough money	136 (51%)	60 (23%)	196 (37%)	**<0.001**
Discussing decision within the family	72 (27%)	61 (24%)	133 (25%)	0.409
Undergoing traditional treatment or home remedies	20 (7%)	15 (6%)	35 (7%)	0.455
Concerned about time away from work/home	20 (7%)	10 (4%)	30 (6%)	0.078
Other delay (volunteered)^a^	8 (3%)	11 (4%)	19 (4%)	--
*No delay*	*37 (14%)*	*32 (12%)*	*69 (13%)*	*0*.*646*
**Perceived delays in reaching hospital after decision**				
Gathering funds	152 (57%)	71 (28%)	223 (42%)	**<0.001**
Busy roads	101 (38%)	70 (27%)	171 (32%)	**0.011**
Arranging an escort	75 (28%)	67 (26%)	142 (27%)	0.621
Arranging a vehicle	63 (23%)	48 (19%)	111 (21%)	0.175
Poor roads	62 (23%)	30 (12%)	92 (17%)	**<0.001**
Distance to hospital	54 (20%)	36 (14%)	90 (17%)	0.062
Too unwell to travel	9 (3%)	12 (5%)	21 (4%)	0.444
Frequent stops	9 (3%)	6 (2%)	15 (3%)	0.481
Slow/no vehicle	2 (1%)	1 (0%)	3 (1%)	0.587
Other delay (volunteered)^b^	7 (3%)	6 (2%)	13 (2%)	--
*No delay*	*62 (23%)*	*97 (36%)*	*159 (30%)*	**<0.001**

^a^ Other sources of delays to the decision volunteered (number volunteering): negative impression of CMCH (6); positive impression of another source (6); unfamiliar with choices for escalation (3); decision-maker unavailable (3); Ramadan (1); another household member unwell at home (1).

^b^ Other sources of delays to transport volunteered: night (8); unsure how to reach hospital (2); arranging leave from employer (1); no one available to care for children (1).

The most commonly cited cause of delay in transport to hospital was the need to gather funds, reported by 42.3% of participants. MPI poor participants encountered this delay more often. MPI poor participants were also more likely to report that poor or busy roads had prolonged their journeys. Means of transport are summarized in **S3 Table**.

### Costs of pre-hospital treatment, and impoverishment

Participants’ estimates of pre-hospital costs are shown in **Table 7**. In many cases, the member of the household with greatest control over spending was unavailable for interview; only respondents who were able to estimate costs are included in this analysis. Equivalent proportions of the poor and non-poor groups were able to estimate expenses (P = 0.164). In both poor and non-poor populations, expenditure was highest for investigations, medication, and medical consumables. Poor participants spent significantly more than non-poor participants on accommodation and transport. Total pre-hospital expenditure consumed a greater proportion of household income for poor participants.

**Table 7:** Estimated pre-hospital expenditure on medical care, days lost from work by household members, and proportion of MPI groups experiencing catastrophic expenditure.

	All			MPI Poor			MPI Non-Poor			Poor *vs*. Non-Poor
Pre-hospital cost by type of expense (Tk)*	*n*	Med	[IQR, Range]	*n*	Med	[IQR, Range]	*n*	Med	[IQR, Range]	P-value^a^
Investigations	428	500	[0–1500, 0–15000]	204	500	[0–1500, 0–6000]	224	1000	[0–1500, 0–15000]	0.610
Medication and medical consumables	353	900	[350–2000, 0–25000]	175	900	[400–2000, 0–10000]	178	900	[275–2000, 10–25000]	0.441
Accommodation (patient and attendants)	421	0	[0–500, 0–6730]	205	0	[0–606, 0–6730]	216	0	[0–325, 0–6500]	**0.005**
Transport	481	450	[200–850, 0–8000]	246	500	[260–1000, 0–8000]	235	350	[140–700, 0–6200]	**<0.001**
Other known expenses	27	200	[200–500, 50–7000]	18	250	[100–500, 50–7000]	9	200	[200–400, 80–600]	0.979
**Total pre-hospital costs**										
All expenses (absolute, Tk)	310	2575	[1050–5500, 20–23500]	152	2750	[1280–5875, 60–2150]	158	2500	[800–5180,20–23500]	0.097
All expenses (% of monthly income)	305	33%	[12%-59%, 0.1%-605%]	149	38%	[22%-71%, 1%-337%]	156	22%	[7%-54%, 0.1%-605%]	**<0.001**
Days of work lost by household	519	6	[4–12, 0–26]	266	7	[4–12, 0–26]	253	6	[3–10, 0–26]	**0.012**
**Pre-hospital costs exceeding thresholds for catastrophic expenditure (*n* = 305)**	***n***	**%**		***n***	**%**		***n***	**%**		
≥ 25% monthly household income	176	58%		101	68%		75	48%		**<0.001**
≥ 40% monthly household income	131	43%		74	50%		57	37%		**0.021**
≥ 100% monthly household income	38	13%		24	16%		14	9%		0.059

*Includes estimates of 0Tk, but excludes those unable to offer an estimate for this category. Therefore, *n* varies between expense categories.

^a^ P-values obtained from Mann-Whitney U test for costs and proportion test for expenditure thresholds.

Prior to hospital admission, a median of 7 days’ work was lost by poor households compared to 6 days’ work lost by non-poor households, (P = 0.012), with a strong skew towards a subset of patients in both groups requiring extensive care from multiple members of the household. This figure combined direct loss of the patient’s days of work and indirect loss by those caring for him or her. The number of participating households that had already faced catastrophic costs by the time of hospital admission was estimated (**Table 7**). We found a high proportion of both poor and non-poor households had already experienced catastophic levels expenditure, with strong evidence of a greater effect on poorer households.

As a further assessment of economic consequences of pre-hospital management of AFI, participants were asked about their households’ means of paying for the costs incurred. The sources of payment are summarized in **Table 8**. The majority of participants in both MPI categories were unable to pay from their household savings alone: overall, 78% had to seek funds from outside of the household, and 63% undertook debts that would require repayment. MPI poor participants were less likely to be able to pay from their own savings (P<0.001), and more likely to take on debts to friends and family, banks, and other moneylenders (P<0.001).

**Table 8:** Sources of payment for expenses arising from illness before arrival at the referral hospital.

	All	MPI Poor	MPI Non-Poor	Poor *vs*. Non-Poor
	*n* = 527	*n* = 269	*n* = 258	
**Means of payment for pre-hospital care**	***n* (%)**	***n* (%)**	***n* (%)**	**P-value**^a^
Own Savings	376 (71%)	171 (64%)	205 (79%)	**<0.001**
Loan from Relatives/Friends	316 (60%)	200 (74%)	116 (45%)	**<0.001**
Gift from Relatives/Friends	124 (24%)	70 (26%)	54 (21%)	0.168
Other Moneylender	92 (17%)	68 (25%)	24 (9%)	**<0.001**
Bank Loans	13 (2%)	13 (5%)	--	**<0.001**
Sale of Property^b^	9 (2%)	6 (2%)	3 (1%)	0.344
Other Source^c^	4 (1%)	--	4 (2%)	--
**Liquidity of payment**				
Unable to pay from own savings alone	410 (78%)	246 (91%)	164 (64%)	**<0.001**
Unable to pay without incurring debts	334 (63%)	213 (79%)	121 (47%)	**<0.001**

^a^P-values from proportion tests, Poor vs. Non-Poor.

^b^ Property sold: land (2); livestock (2); tea shop (1); rickshaw (1); tree (1); gold ornaments (1); television (1).

^c^ Other sources of payment volunteered: payment by employer (4).

## Discussion

Our survey of patients with acute febrile illnesses identified important delays, obstacles, and costs associated with multidimensional poverty, arising between onset of symptoms and arrival at hospital. We found that for adults from multidimensionally poor households, median time to arrival at the referral hospital was approximately one day longer than for those from non-poor households. This effect was not observed for children, among whom pre-hospital delays were of similar magnitude for poor and non-poor households. Given that timely initiation of properly directed antimicrobial therapy is a well-established determinant of survival in many severe infectious diseases, this delay poses a substantial clinical risk [13–16]. We observed a higher case fatality among patients from poorer households, and while a multitude of factors undoubtedly contributed to this excess mortality, it underlines the urgency of reducing delays wherever possible.

Participants from all backgrounds recognized the need for help from outside the household early in the course of the AFI. However, even in this initial step, MPI poor adults took longer than non-poor adults to seek help (with equivalent delays among poor and non-poor children). This implies that participants recognized the potential seriousness of AFI and its responsiveness to treatment, but that barriers to care associated with poverty arise from an early stage. The interval from first seeking help to the household’s decision to escalate care to the referral hospital was longer for adults from poor households compared to non-poor. This may reflect higher utilization of qualified private doctors by non-poor adults, compared to poor adults and children, who were more likely to consult unqualified practitioners. It is possible that qualified doctors were more likely to recognize the potential seriousness of AFI, and warn patients of the possible need for escalation.

The differential effect of poverty on different age strata has more than one plausible explanation. Households may perceive childhood fever as more dangerous and urgent—and so prioritize the timely care of children regardless of cost—reducing the impact of poverty on delays for children of both poor and non-poor households. Alternatively, non-poor adults may be prioritized, and encounter the fewest delays because their households are the most willing and able to pay. Both patterns of prioritization have been reported in care-seeking for acute febrile illnesses in different contexts [37, 38]. Given the complexity of care-seeking behaviour, we hope to gain a clearer understanding of the decision-making process, and the differential effects of poverty at different ages, through an ongoing qualitative study of this population. Reducing barriers to care stands to benefit all age strata, and may help to prevent the catastrophic levels of expenditure seen among febrile adults and children. Therefore, we would recommend addressing barriers to care for all ages, though the greatest gains of equity may be seen among adults.

Our findings suggest that the best opportunity to reduce pre-hospital delays is during the period in which patients are seeking help outside the home, before the decision to escalate to the referral hospital is made. It is important, therefore, to analyze where healthcare is sought, and what routes patients follow when an illness progresses. In keeping with international studies, most patients followed a pattern of escalation from sources nearest the home, most accessible, and least expensive, toward greater professional expertise and more intensive management [33]. We observed few consultations with non-allopathic traditional healers, either by MPI poor or non-poor households. This corroborates previous observations that the population recognizes AFIs as amenable to allopathic treatment [18, 28, 39].

In most cases, a private sector hierarchy—from shop to private allopath/doctor to hospital—supplanted the public sector hierarchy from clinic to health complex to hospital. Despite increased government investment in public-sector healthcare, serial community surveys have shown falling perception of quality and satisfaction, with highest dissatisfaction among the poorest households [28]. Patients have identified under-staffing, poor physical facilities, behavior of service providers, and inability to provide essential medicines directly as causes of this low opinion. Healthcare providers have noted consistent problems with drug procurement and supply. Absenteeism is common—reported at 40% for doctors at health complexes nationwide, rising higher in poorer areas [40, 41]. The majority of patients attending public sector services are required to pay for medicines from private vendors due to depletion of essential drug stocks, and many experience pressure to pay unofficial fees to members of staff [28, 42–44].

Participants perceived financial constraints as a major cause of delays in seeking healthcare. These constraints disproportionately affected poorer households, but were a problem for less poor households as well. AFI can be financially disastrous to households already afflicted by poverty, and that unmanageable costs begin to accrue early in the patient journey. At the point of arrival at the referral hospital, a majority of households had already extended beyond their savings to pay for medical care, incurred debts that would require repayment, and reached the point of catastrophic expenditure. All of these consequences were more common for poorer households. Expenses usually rise steeply after admission, and families face the added burdens of time away from work and accommodation far from home for those who accompany the patient [32]. The decision to seek medical care poses a considerable risk of financial ruin, particularly for those already afflicted by multidimensional poverty [32, 45]. Addressing this risk may reduce delays due to reluctance to take on this burden, and those due to the need to gather funds.

Several constructive recommendations for the private and public sectors can be drawn from this investigation. Our findings imply that there is scope to improve the accessibility and quality of the government sector, so that its well-structured but under-utilized hierarchy becomes a desirable option for patients with AFI. Addressing absenteeism, ensuring that patients are aware of their rights to free essential drugs and services, and extending government subsidies to the dispensation of more drugs and ancillary care will improve both the quality and the uptake of public sector facilities closer to home, and reduce costs and delays for patients [28, 41].

Educating informal private sector providers in the immediate management of AFIs—particularly in malaria-endemic parts of the Division—might improve such practices, and save lives [10, 22]. Engagement initiatives should offer education on the recognition of warning signs (such as reduced level of consciousness) that should prompt a provider to refer a patient to a qualified doctor or hospital. New tools such as RDTs for malaria and other infections have the potential to give para-professional providers a limited but important role in diagnosis and treatment algorithms. This could help to integrate public and private services, and streamline what is currently a long and costly process of serial consultation. As the first responders to many potentially fatal illnesses, providers in this sector have a critical opportunity to intervene.

Provision of insurance schemes to the poor should be promoted. Access to health insurance will encourage early medical consultation with qualified practitioners—without the need to gather funds and take on debts. Insurance also increases capacity to respond to high costs of in-patient care when this is necessary [46, 47]. Demand-side financing has been introduced to promote maternal health in rural Bangladesh, by providing credit vouchers in advance of need for health services. This appears to reduce cost and increase consultations with appropriate practitioners [48]. Extending such programs may help to reduce household expenditure and promote early consultation and escalation. Previous studies of insurance schemes in Bangladesh have identified benefits in terms of access to basic and preventative healthcare, but found that insured households are not protected from the catastrophic expenses of severe illness, necessitating hospital admission, where the costs of interventions and supportive care are not covered, and can escalate steeply [47]. Extending such programmes to cover high-cost care would help to mitigate the financial ruin that can stem from acute illness.

In a setting where hospital admissions pose considerable financial strain, and where in-patient demand exceeds provision, recommendations that leads to too many hospital admissions could be as dangerous as those that lead to too few [32]. An optimal community service would prevent a majority of hospital admissions by providing diagnosis and treatment early, while fast-tracking those cases that require urgent escalation.

In the long term, social changes that address inequities in assets, living standards, education, and chronic health in Bangladesh will have positive consequences for the pre-hospital management of AFIs and other causes of preventable morbidity and mortality. There is evidence from longitudinal studies that integrated development interventions have already had a positive impact on health seeking as well as health outcomes elsewhere in Bangladesh [49, 50].

Our study has several important limitations. The potential for selection bias is acknowledged. The goal of daily recruitment of one patient from the adult medical wards and one from the paediatric medical wards was aimed at ensuring a systematic sample through the year, but more consideration could have been given to ensuring a random selection among those febrile patients admitted during a 24-hour period. Failure to randomize among each day’s admissions may have led to under-sampling of participants who were admitted for a short period, with rapid discharge or clinical deterioration and death. It is likely that such under-sampling would be non-differential, affecting poor and non-poor groups equally; this would bias the results toward the null hypothesis, and lead to a more conservative estimate of difference between poor and non-poor populations.

This study does not capture those febrile illnesses that did not lead to hospitalization—either because adequate care was obtained elsewhere, or because barriers to hospital care were insurmountable. It is likely that this study under-represents those residents of the Chittagong Division who are worst afflicted by multidimensional poverty, as well as those who are geographically most remote, since these groups might never reach the referral hospital.

The potential for misclassification bias is also acknowledged, with regard to ascertainment of MPI status in the context of acute illness. While all other components of MPI can be ascertained based on household characteristics preceding the illness, nutrition status of the household must be measured at the time of interview, and could potentially be affected by rapid weight loss as a consequence of AFI. This could have led to over-estimation of malnutrition and misclassification of some non-poor households as poor. There were 39 cases in which the participant’s low nutritional status could have affected MPI sufficiently to change classification. While it is unlikely that all patients experienced marked weight loss, in this scenario the maximum rate of misclassification due to acute weight loss was 7%. Had these cases been re-classified, the assessment of the primary outcome measures would have been unchanged.

Acknowledging these caveats, our study contributes to a more complete understanding of care-seeking behavior and the obstacles posed by poverty. This understanding is essential to confronting the complex relationship between poverty and illness, and prioritizing interventions that address these two inseparable problems.

## Supporting Information

S1 Table Transitions between sources of healthcare undertaken by participants with AFI. (DOCX)

S2 Table Routes to the referral hospital, undertaken by 527 patients with AFI, beginning with the first source of help outside the home, and ranked for frequency of utilization. (DOCX)

S3 Table Means of transport to the referral hospital. (DOCX)
