# Supplementary material for: The Relationship between Poverty and Healthcare Seeking among Patients Hospitalized with Acute Febrile Illnesses in Chittagong, Bangladesh
Source: PLoS One. 2016 Apr 7;11(4):e0152965. doi: 10.1371/journal.pone.0152965 (PMC4824474; doi:10.1371/journal.pone.0152965)
Supplement: S1 Table — (DOCX) [file pone.0152965.s001.docx]

**Table S1: Transitions between sources of healthcare undertaken by participants with AFI.**

| **All Patients** | **TO** | **Shop/**  **Pharmacy** | **Private Doctor** | **Allopath** | **GHC** | **Gov Hospital** | **Traditional Healer** | **Private Hospital** | **Friends/**  **Relatives** | **Other Source** | **REFERRAL HOSPITAL** | **TOTAL** |
| --- | --- | --- | --- | --- | --- | --- | --- | --- | --- | --- | --- | --- |
| **FROM** | HOME | 257 | 111 | 59 | 32 | 10 | 16 | 8 | 8 | 1 | 25 | 527 |
|  | Shop/Pharmacy | . | 75 | 80 | 37 | 16 | 11 | 7 | . | . | 39 | 265 |
|  | Private Doctor | . | 18 | . | 30 | 31 | 1 | 14 | . | . | 131 | 225 |
|  | Allopath | 2 | 2 | . | 11 | 9 | . | 8 | 2 | 1 | 110 | 145 |
|  | GHC | . | 7 | 2 | 1 | 8 | . | 7 | . | 1 | 99 | 125 |
|  | Gov Hospital | . | . | 1 | 1 | . | . | 1 | . | . | 74 | 77 |
|  | Trad Healer | 1 | 9 | 1 | 9 | . | . | . | . | 1 | 7 | 28 |
|  | Private Hospital | 1 | 3 | 2 | 4 | 2 | . | . | . | . | 33 | 45 |
|  | Friends/Relatives | 4 | . | . | 1 | . | . | . | . | . | 5 | 10 |
|  | Other Source | 1 | . | . | . | . | . | . | . | . | 4 | 5 |
|  |  |  |  |  |  |  |  |  |  |  |  |  |
| **MPI Poor** | **TO** | **Shop/**  **Pharmacy** | **Private Doctor** | **Allopath** | **GHC** | **Gov Hospital** | **Traditional Healer** | **Private Hospital** | **Friends/**  **Relatives** | **Other Source** | **REFERRAL HOSPITAL** | **TOTAL** |
| **FROM** | HOME | 139 | 42 | 35 | 20 | 6 | 9 | 4 | 5 | . | 9 | 269 |
|  | Shop/Pharmacy | . | 36 | 47 | 23 | 7 | 7 | 3 | . | . | 21 | 144 |
|  | Private Doctor | . | 8 | . | 14 | 14 | . | 6 | . | . | 57 | 99 |
|  | Allopath | 2 | . | . | 7 | 7 | . | 6 | . | 1 | 63 | 86 |
|  | GHC | . | 5 | . | . | 5 | . | 4 | . | 1 | 55 | 70 |
|  | Gov Hospital | . | . | 1 | . | . | . | 1 | . | . | 39 | 41 |
|  | Trad Healer | . | 7 | 1 | 4 | . | . | . | . | 1 | 3 | 16 |
|  | Private Hospital | 1 | 1 | 2 | 1 | 2 | . | . | . | . | 17 | 24 |
|  | Friends/Relatives | 3 | . | . | 1 | . | . | . | . | . | 1 | 5 |
|  | Other Source | . | . | . | . | . | . | . | . | . | 4 | 4 |
|  |  |  |  |  |  |  |  |  |  |  |  |  |
| **MPI Non-Poor** | **TO** | **Shop/**  **Pharmacy** | **Private Doctor** | **Allopath** | **GHC** | **Gov Hospital** | **Traditional Healer** | **Private Hospital** | **Friends/**  **Relatives** | **Other Source** | **REFERRAL HOSPITAL** | **TOTAL** |
| **FROM** | HOME | 118 | 69 | 24 | 12 | 4 | 7 | 4 | 3 | 1 | 16 | 258 |
|  | Shop/Pharmacy | . | 39 | 33 | 14 | 9 | 4 | 4 | . | . | 18 | 121 |
|  | Private Doctor | . | 10 | . | 16 | 17 | 1 | 8 | . | . | 74 | 126 |
|  | Allopath | . | 2 | . | 4 | 2 | . | 2 | 2 | . | 47 | 59 |
|  | GHC | . | 2 | 2 | 1 | 3 | . | 3 | . | . | 44 | 55 |
|  | Gov Hospital | . | . | . | 1 | . | . | . | . | . | 35 | 36 |
|  | Trad Healer | 1 | 2 | . | 5 | . | . | . | . | . | 4 | 12 |
|  | Private Hospital | . | 2 | . | 3 | . | . | . | . | . | 16 | 21 |
|  | Friends/Relatives | 1 | . | . | . | . | . | . | . | . | 4 | 5 |
|  | Other Source | 1 | . | . | . | . | . | . | . | . | . | 1 |

| **MPI Poor**  **Adults** | **TO** | **Shop/**  **Pharmacy** | **Private Doctor** | **Allopath** | **GHC** | **Gov Hospital** | **Traditional Healer** | **Private Hospital** | **Friends/**  **Relatives** | **Other Source** | **REFERRAL HOSPITAL** | **TOTAL** |
| --- | --- | --- | --- | --- | --- | --- | --- | --- | --- | --- | --- | --- |
| **FROM** | HOME | 30 | 13 | 25 | 10 | 4 | 4 | 3 | . | . | 9 | 98 |
|  | Shop/Pharmacy | . | 11 | 5 | 7 | 2 | . | 1 | . | 1 | 5 | 32 |
|  | Private Doctor | . | 1 | . | . | 6 | . | 1 | . | . | 21 | 29 |
|  | Allopath | 1 | . | . | 4 | 3 | . | 2 | . | 1 | 20 | 31 |
|  | GHC | . | 1 | . | . | 1 | . | 2 | . | . | 18 | 22 |
|  | Gov Hospital | . | . | . | . | . | . | . | . | . | 17 | 17 |
|  | Trad Healer | . | 3 | . | . | . | . | . | . | . | 1 | 4 |
|  | Private Hospital | 1 | . | 1 | 1 | 1 | . | . | . | . | 5 | 9 |
|  | Friends/Relatives | . | . | . | . | . | . | . | . | . | . | . |
|  | Other Source | . | . | . | . | . | . | . | . | . | 2 | 2 |
|  |  |  |  |  |  |  |  |  |  |  |  |  |
| **MPI Non-Poor Adults** | **TO** | **Shop/**  **Pharmacy** | **Private Doctor** | **Allopath** | **GHC** | **Gov Hospital** | **Traditional Healer** | **Private Hospital** | **Friends/**  **Relatives** | **Other Source** | **REFERRAL HOSPITAL** | **TOTAL** |
| **FROM** | HOME | 47 | 49 | 21 | 5 | 1 | 3 | 3 | 2 | . | 13 | 144 |
|  | Shop/Pharmacy | . | 9 | 7 | 10 | 8 | 1 | 2 | . | . | 10 | 47 |
|  | Private Doctor | . | 6 | . | 5 | 12 | . | 3 | . | . | 42 | 68 |
|  | Allopath | . | 2 | . | 2 | 1 | . | . | 2 | . | 22 | 29 |
|  | GHC | . | . | 1 | . | 2 | . | 1 | . | . | 19 | 23 |
|  | Gov Hospital | . | . | . | . | . | . | . | . | . | 24 | 24 |
|  | Trad Healer | . | 1 | . | 1 | . | . | . | . | . | 2 | 4 |
|  | Private Hospital | . | 1 | . | . | . | . | . | . | . | 8 | 9 |
|  | Friends/Relatives | . | . | . | . | . | . | . | . | . | 4 | 4 |
|  | Other Source | . | . | . | . | . | . | . | . | . | . | . |

| **MPI Poor**  **Children** | **TO** | **Shop/**  **Pharmacy** | **Private Doctor** | **Allopath** | **GHC** | **Gov Hospital** | **Traditional Healer** | **Private Hospital** | **Friends/**  **Relatives** | **Other Source** | **REFERRAL HOSPITAL** | **TOTAL** |
| --- | --- | --- | --- | --- | --- | --- | --- | --- | --- | --- | --- | --- |
| **FROM** | HOME | 109 | 29 | 10 | 10 | 2 | 5 | 1 | 5 | . | . | 171 |
|  | Shop/Pharmacy | . | 25 | 42 | 16 | 5 | 7 | 2 | . | . | 16 | 113 |
|  | Private Doctor | . | 7 | . | 14 | 8 | . | 5 | . | . | 36 | 70 |
|  | Allopath | 1 | . | . | 3 | 4 | . | 4 | . | . | 43 | 55 |
|  | GHC | . | 4 | . | . | 4 | . | 2 | . | 1 | 37 | 48 |
|  | Gov Hospital | . | . | 1 | . | . | . | 1 | . | . | 22 | 24 |
|  | Trad Healer | . | 4 | 1 | 4 | . | . | . | . | 1 | 2 | 12 |
|  | Private Hospital | . | 1 | 1 | . | 1 | . | . | . | . | 12 | 15 |
|  | Friends/Relatives | 3 | . | . | 1 | . | . | . | . | . | 1 | 5 |
|  | Other Source | . | . | . | . | . | . | . | . | . | 2 | 2 |
|  |  |  |  |  |  |  |  |  |  |  |  |  |
| **MPI Non-Poor Children** | **TO** | **Shop/**  **Pharmacy** | **Private Doctor** | **Allopath** | **GHC** | **Gov Hospital** | **Traditional Healer** | **Private Hospital** | **Friends/**  **Relatives** | **Other Source** | **REFERRAL HOSPITAL** | **TOTAL** |
| **FROM** | HOME | 71 | 20 | 3 | 7 | 3 | 4 | 1 | 1 | 1 | 3 | 114 |
|  | Shop/Pharmacy | . | 30 | 26 | 4 | 1 | 3 | 2 | . | . | 8 | 74 |
|  | Private Doctor | . | 5 | . | 11 | 5 | 1 | 5 | . | . | 32 | 59 |
|  | Allopath | . | . | . | 2 | 1 | . | 2 | . | . | 25 | 30 |
|  | GHC | . | 2 | 1 | 1 | 1 | . | 2 | . | . | 26 | 33 |
|  | Gov Hospital | . | . | . | 1 | . | . | . | . | . | 10 | 11 |
|  | Trad Healer | 1 | 1 | . | 4 | . | . | . | . | . | 2 | 8 |
|  | Private Hospital | . | 1 | . | 3 | . | . | . | . | . | 8 | 12 |
|  | Friends/Relatives | 1 | . | . | . | . | . | . | . | . | . | 1 |
|  | Other Source | 1 | . | . | . | . | . | . | . | . | . | 1 |
