# Supplementary material for: The Relationship between Poverty and Healthcare Seeking among Patients Hospitalized with Acute Febrile Illnesses in Chittagong, Bangladesh
Source: PLoS One. 2016 Apr 7;11(4):e0152965. doi: 10.1371/journal.pone.0152965 (PMC4824474; doi:10.1371/journal.pone.0152965)
Supplement: S2 Table — (DOCX) [file pone.0152965.s002.docx]

**Table S2: Routes to the referral hospital, undertaken by 527 patients with AFI, beginning with the first source of help outside the home, and ranked for frequency of utilization.**

| **Sequence Rank** | **Care Sequence ^a^** | **Non-Poor** | **Poor** | **All** | | **Cumulative** |
| --- | --- | --- | --- | --- | --- | --- |
|  |  | ***n*** | ***n*** | ***n*** | **%** | **%** |
| 1 | D-R | 42 | 22 | 64 | 12.1% | 12.1% |
| 2 | S-U-R | 28 | 35 | 63 | 12.0% | 24.1% |
| 3 | U-R | 17 | 23 | 40 | 7.6% | 31.7% |
| 4 | S-R | 18 | 21 | 39 | 7.4% | 39.1% |
| 5 | S-D-R | 18 | 20 | 38 | 7.2% | 46.3% |
| 6 | S-C-R | 9 | 18 | 27 | 5.1% | 51.4% |
| 7 | R | 16 | 9 | 25 | 4.7% | 56.2% |
| 8 | C-R | 9 | 16 | 25 | 4.7% | 60.9% |
| 9 | D-G-R | 10 | 5 | 15 | 2.8% | 63.8% |
| 10 | S-D-C-R | 7 | 8 | 15 | 2.8% | 66.6% |
| 11 | S-G-R | 9 | 5 | 14 | 2.7% | 69.3% |
| 12 | S-D-G-R | 6 | 5 | 11 | 2.1% | 71.3% |
| 13 | D-D-R | 5 | 4 | 9 | 1.7% | 73.1% |
| 14 | G-R | 3 | 6 | 9 | 1.7% | 74.8% |
| 15 | D-P-R | 4 | 4 | 8 | 1.5% | 76.3% |
| 16 | D-C-R | 6 | 1 | 7 | 1.3% | 77.6% |
| 17 | S-P-R | 3 | 2 | 5 | 0.9% | 78.6% |
| 18 | T-C-R | 3 | 2 | 5 | 0.9% | 79.5% |
| 19 | U-C-R | 1 | 4 | 5 | 0.9% | 80.5% |
| 20 | P-R | 3 | 1 | 4 | 0.8% | 81.2% |
| 21 | S-D-D-R | 2 | 2 | 4 | 0.8% | 82.0% |
| 22 | S-U-G-R | 1 | 3 | 4 | 0.8% | 82.7% |
| 23 | S-U-P-R | 1 | 3 | 4 | 0.8% | 83.5% |
| 24 | U-G-R | 1 | 3 | 4 | 0.8% | 84.3% |
| 25 | T-D-R |  | 4 | 4 | 0.8% | 85.0% |
| 26 | F-R | 2 | 1 | 3 | 0.6% | 85.6% |
| 27 | S-C-P-R | 2 | 1 | 3 | 0.6% | 86.1% |
| 28 | S-D-P-R | 2 | 1 | 3 | 0.6% | 86.7% |
| 29 | T-R | 2 | 1 | 3 | 0.6% | 87.3% |
| 30 | U-F-R | 2 | . | 2 | 0.4% | 87.7% |
| 31 | S-C-D-R | 1 | 1 | 2 | 0.4% | 88.0% |
| 32 | S-C-G-R | 1 | 1 | 2 | 0.4% | 88.4% |
| 33 | S-T-C-R | 1 | 1 | 2 | 0.4% | 88.8% |
| 34 | S-T-R | 1 | 1 | 2 | 0.4% | 89.2% |
| 35 | S-U-C-R | 1 | 1 | 2 | 0.4% | 89.6% |
| 36 | C-D-R | . | 2 | 2 | 0.4% | 89.9% |
| 37 | C-G-R | . | 2 | 2 | 0.4% | 90.3% |
| 38 | D-C-P-R | . | 2 | 2 | 0.4% | 90.7% |
| 39 | F-S-U-R | . | 2 | 2 | 0.4% | 91.1% |
| 40 | C-C-R | 1 | . | 1 | 0.2% | 91.3% |
| 41 | C-P-R | 1 | . | 1 | 0.2% | 91.5% |
| 42 | C-U-R | 1 | . | 1 | 0.2% | 91.7% |
| 43 | D-D-D-R | 1 | . | 1 | 0.2% | 91.8% |
| 44 | D-P-C-R | 1 | . | 1 | 0.2% | 92.0% |
| 45 | F-S-D-R | 1 | . | 1 | 0.2% | 92.2% |
| 46 | G-C-R | 1 | . | 1 | 0.2% | 92.4% |
| 47 | O-S-P-D-R | 1 | . | 1 | 0.2% | 92.6% |
| 48 | P-D-R | 1 | . | 1 | 0.2% | 92.8% |
| 49 | S-C-U-R | 1 | . | 1 | 0.2% | 93.0% |
| 50 | S-D-D-C-G-R | 1 | . | 1 | 0.2% | 93.2% |
| 51 | S-D-D-P-G-R | 1 | . | 1 | 0.2% | 93.4% |
| 52 | S-D-T-R | 1 | . | 1 | 0.2% | 93.5% |
| 53 | S-T-C-D-R | 1 | . | 1 | 0.2% | 93.7% |
| 54 | S-T-D-C-R | 1 | . | 1 | 0.2% | 93.9% |
| 55 | S-U-P-C-R | 1 | . | 1 | 0.2% | 94.1% |
| 56 | T-D-G-R | 1 | . | 1 | 0.2% | 94.3% |
| 57 | T-S-U-C-R | 1 | . | 1 | 0.2% | 94.5% |
| 58 | U-C-G-R | 1 | . | 1 | 0.2% | 94.7% |
| 59 | U-D-C-R | 1 | . | 1 | 0.2% | 94.9% |
| 60 | U-D-R | 1 | . | 1 | 0.2% | 95.1% |
| 61 | D-C-G-R | . | 1 | 1 | 0.2% | 95.3% |
| 62 | D-C-O-R | . | 1 | 1 | 0.2% | 95.4% |
| 63 | D-G-P-R | . | 1 | 1 | 0.2% | 95.6% |
| 64 | D-P-G-R | . | 1 | 1 | 0.2% | 95.8% |
| 65 | F-C-R | . | 1 | 1 | 0.2% | 96.0% |
| 66 | F-S-T-R | . | 1 | 1 | 0.2% | 96.2% |
| 67 | P-C-R | . | 1 | 1 | 0.2% | 96.4% |
| 68 | P-U-G-R | . | 1 | 1 | 0.2% | 96.6% |
| 69 | P-U-R | . | 1 | 1 | 0.2% | 96.8% |
| 70 | S-C-D-G-R | . | 1 | 1 | 0.2% | 97.0% |
| 71 | S-G-U-R | . | 1 | 1 | 0.2% | 97.2% |
| 72 | S-O-R | . | 1 | 1 | 0.2% | 97.3% |
| 73 | S-P-D-D-R | . | 1 | 1 | 0.2% | 97.5% |
| 74 | S-T-C-D-G-R | . | 1 | 1 | 0.2% | 97.7% |
| 75 | S-T-D-D-R | . | 1 | 1 | 0.2% | 97.9% |
| 76 | S-T-D-G-R | . | 1 | 1 | 0.2% | 98.1% |
| 77 | S-T-O-R | . | 1 | 1 | 0.2% | 98.3% |
| 78 | S-U-C-G-R | . | 1 | 1 | 0.2% | 98.5% |
| 79 | S-U-O-R | . | 1 | 1 | 0.2% | 98.7% |
| 80 | T-D-C-R | . | 1 | 1 | 0.2% | 98.9% |
| 81 | T-U-P-R | . | 1 | 1 | 0.2% | 99.1% |
| 82 | U-C-P-G-R | . | 1 | 1 | 0.2% | 99.2% |
| 83 | U-P-R | . | 1 | 1 | 0.2% | 99.4% |
| 84 | U-P-S-U-R | . | 1 | 1 | 0.2% | 99.6% |
| 85 | U-S-C-R | . | 1 | 1 | 0.2% | 99.8% |
| 86 | U-S-G-R | . | 1 | 1 | 0.2% | 100.0% |

^a^ S = shop or pharmacy, D = private doctor, U = allopathic practitioner, C = government health complex/clinic, G = government hospital; T = traditional healer, P = private hospital, F = friends or relatives, O = other source, R = referral hospital (CMCH).
