# Supplementary material for: The Relationship between Poverty and Healthcare Seeking among Patients Hospitalized with Acute Febrile Illnesses in Chittagong, Bangladesh
Source: PLoS One. 2016 Apr 7;11(4):e0152965. doi: 10.1371/journal.pone.0152965 (PMC4824474; doi:10.1371/journal.pone.0152965)
Supplement: S3 Table — (DOCX) [file pone.0152965.s003.docx]

**Table S3. Means of transport to the referral hospital.**

| **Means of Transport** | **All** | | **MPI Poor** | | **MPI Non-Poor** | | **Poor *vs.* Non-Poor** |
| --- | --- | --- | --- | --- | --- | --- | --- |
| **(multiple answers permitted)** | ***n* = 527** | | ***n* = 269** | | ***n* = 258** | |  |
|  | ***n*** | **(%)** | ***n*** | **(%)** | ***n*** | **(%)** | **P-value^a^** |
| CNG motorized rickshaw | 339 | (64.3) | 155 | (57.6) | 184 | (71.3) | **0.001** |
| Rickshaw | 191 | (36.2) | 114 | (42.4) | 77 | (29.8) | **0.003** |
| Foot | 144 | (27.3) | 101 | (37.5) | 43 | (16.7) | **<0.001** |
| Bus | 112 | (21.3) | 77 | (28.6) | 35 | (13.6) | **<0.001** |
| Ambulance | 54 | (10.2) | 29 | (10.8) | 25 | (9.7) | **0.006** |
| Boat | 8 | (1.5) | 7 | (2.6) | 1 | (0.4) | 0.069 |
| Car | 5 | (0.9) | 1 | (0.4) | 4 | (1.6) | 0.208 |
| *Other* | 2 | (0.4) | 1^b^ | (0.4) | 1^c^ | (0.4) | 1.000 |
| *Any Motorized Transport* | 468 | (88.8) | 239 | (88.9) | 229 | (88.8) | 0.249 |

^a^ Comparisons by Fisher’s exact test. ^b^ Transport by unknown means. ^c^ Train.
